# IL-18BP Improves Early Graft Function and Survival in Lewis–Brown Norway Rat Orthotopic Liver Transplantation Model

**DOI:** 10.3390/biom12121801

**Published:** 2022-12-01

**Authors:** Qiang Meng, Weikang Wu, Wenjie Zhang, Juzheng Yuan, Long Yang, Xuan Zhang, Kaishan Tao

**Affiliations:** 1 Department of Hepatobiliary Surgery, Xi-Jing Hospital, Fourth Military Medical University, Xi’an 710032, China; 2 Chinese Education Ministry’s Key Laboratory of Western Resources and Modern Biotechnology, Key Laboratory of Biotechnology Shaanxi Province, College of Life Sciences, Northwest University, Xi’an 710032, China

**Keywords:** orthotopic liver transplantation, liver xenotransplantation, IL-18 binding protein, acute vascular rejection, natural killer cell

## Abstract

Interleukin-18 (IL-18) can effectively activate natural killer (NK) cells and induce large concentrations of interferon-γ (IFN-γ). In healthy humans, IL-18 binding protein (IL-18BP) can inhibit the binding of IL-18 to IL-18R and counteract the biological action of IL-18 due to its high concentration and high affinity, thus preventing the production of IFN-γ and inhibiting NK-cell activation. Through previous studies and the phenomena observed by our group in pig–non-human primates (NHPs) liver transplantation experiments, we proposed that the imbalance in IL-18/IL-18BP expression upon transplantation encourages the activation, proliferation, and cytotoxic effects of NK cells, ultimately causing acute vascular rejection of the graft. In this research, we used Lewis–Brown Norway rat orthotopic liver transplantation (OLTx) as a model of acute vascular rejection. AAV8-Il18bp viral vectors as gene delivery vehicles were constructed for gene therapy to overexpress IL-18BP and alleviate NK-cell rejection of the graft after transplantation. The results showed that livers overexpressing IL-18BP had reduced damage and could function longer after transplantation, effectively improving the survival time of the recipients.

## 1. Introduction

Patients who suffer from end-stage liver disease (ESLD) and acute hepatic failure (AHF) benefit well from liver transplantation, but it is severely hampered by a lack of donor organs, and many patients pass away while waiting [1]. Xenotransplantation is an effective treatment that solves organ shortages and effectively prolongs patient life [2,3]. Previous studies showed that knocking out genes that mainly induce rejection in pigs could effectively alleviate hyperacute rejection of the transplant and prolong donor survival, but it is not yet sufficient for clinical treatment [4,5,6,7]. When humoral rejection was attenuated, cellular rejection was prominently demonstrated. In pathological and histological studies after transplantation, it was found that xenograft infiltration mainly involved NK cells [8,9] and macrophages, and dendritic cells also contributed to immunological tolerance and immunomodulation of immune rejection. Host monocytes [10] and NK cells [11,12] have been identified as the main mediators of delayed allograft rejection in several in vivo and in vitro investigations, and after these cells firmly adhere to the allograft vascular endothelium, they can perform their effector functions, including the production of proinflammatory factors, the generation of adhesion molecules for endothelial cells, and, ultimately, the mediation of endothelial cell lysis.

It was originally discovered that the cytokine IL-18 cooperated with IL-12 to generate IFN-γ in pathogen-infected mice [13]. IL-18 has a major pathophysiological function in several disorders, including coronavirus disease 2019 (COVID-19) [14], given its significant proinflammatory activity and pleiotropic features.

In healthy humans, serum levels of IL-18BP are approximately 20 times higher than IL-18, and this factor has a very high affinity compared to IL-18R [15]. By functioning as a particular receptor protein targeting IL-18 in reaction to its release into the blood, IL-18BP limits the ability of IL-18 to bind to IL-18R and inhibits its biological function, reducing the generation of IFN-γ and restricting the responses of Th1 as well as NK cells [16,17]. More interestingly, IL-18-inducing IFN-γ secretion upregulates IL-18BP expression, thereby maintaining the balance of IL-18/IL-18BP in vivo [18]. Numerous investigations have demonstrated that certain inflammatory illnesses [19,20,21] have significantly raised IL-18 levels in the serum. Mice lacking IL-18BP exhibit more severe symptoms in various illness models. When clinical trials for the management of plaque psoriasis [22], rheumatoid arthritis [23,24], and adult-onset Still’s disease [25] were conducted, exogenous IL-18BP therapy successfully lowered the pathogenic response and showed positive safety and efficacy indications. It is beneficial to treat inflammation-related illnesses by regulating IL-18BP to reduce the strong proinflammatory response of IL-18 [26,27].

In a previous study, our group concluded that the abnormal concentration of IL-18 and the dysregulated ratio of IL-18BP promoted NK-cell infiltration in tissues and mediated immune rejection, as determined by serum cytokine analysis and tissue analysis after pig–NHPs allogeneic liver transplantation, which resulted in acute vascular rejection. In the current study, pig–NHPs xenograft surgery could not be used as a routine research model because of the difficulty and cost. Therefore, to explore acute liver transplant rejection and immunological tolerance in allogeneic rats, we determined the Lewis–Brown Norway inbred rat orthotopic liver transplantation (OLTx) to serve as the perfect animal model [28]. We explored how the IL-18/IL-18BP ratio affected the acute vascular rejection (AVR) of grafts in a LEW-BN rat OLTx model based on previous research results.

## 2. Materials and Methods

### 2.1. Adeno-Associated Virus-8 (AAV-8) Construction

Genetic therapy has been used to cure a number of human disorders by using adeno-associated virus (AAV) vectors [29]. AAV-8 preferentially infects hepatocytes [30,31]. An AAV-8 delivery system was used to overexpress IL-18BP in hepatocytes of LEW rats. The AAV-8 delivery system was developed by Hanbio (Shanghai, China). The titers of the vector genome were determined by RT-PCR and vector-specific primers.

### 2.2. Animals

Male adult Lewis RT1**^ǀ^** (LEW) rats weighing 200–220 g and male adult Brown Norway RT1^n^ (BN) rats weighing 220–240 g were utilized as liver donors and recipients, respectively (Beijing Vital River Laboratory Animal Technology Co., Ltd, Beijing, China). Before and after the experiments, the rats had unlimited access to food and water, and they were given at least 5 days to become acquainted with the animal research facility. The Laboratory Animal Welfare and Ethics Committee of Animal Laboratory Center, Fourth Military Medical University approved the animal study (No. IACUC-20220714).

### 2.3. Tail Vein Injection of AAV-8

To successfully deliver genes to donor rats, 200 μL of virus at a titer of 1.0 × 10^12^ vg/mL AAV8-Il18bp or AAV8-Luc was injected via the tail vein 3 weeks before OLTx. To confirm that AAV-8 could indeed infect rat liver tissue, we performed intravital optical imaging 3 weeks after viral infection. For living animal imaging, D-Luciferin potassium salt (ST196, 15 mg/mL stock in D-PBS, Beyotime, China; 150 μL/g) was administered intraperitoneally to the rats 10 min before imaging began. The rats were put in the isoflurane induction chamber five minutes before imaging and moved to the IVIS imaging chamber after being thoroughly anesthetized. After successfully infecting the liver with AAV-8, the entire liver was taken out and used as a transplant.

### 2.4. Surgery for Organ Transplantation

The animals were administered under isoflurane anesthesia for the procedures on both donors and recipients. OLTx was performed without hepatic artery reconstruction following the method reported by Kamada and Calne [32]. Through an abdominal aorta catheter, 40 mL of lactated Ringer’s solution (4 °C) was infused into the livers. Additionally, the removed grafts were maintained in lactated Ringer’s solution (4 °C). Cold ischemia lasted for less than 90 min. The anhepatic phase lasted for just 16 min. Immunosuppressive medications were not given out. According to earlier research, BN patients who received liver allografts from LEW rats had a median survival duration < 14 d [28,33]. For the sham group, the abdomen of LEW rats infected with AAV8-Luc were opened and closed.

### 2.5. Acquisition of Blood and Tissue

Seven days after OLTx, blood was collected in heparinized whole blood test tubes immediately following the abdominal incision. The serum was separated from whole blood and kept cold at −80 °C. Rats were slaughtered at the same time, and the entire liver tissues were taken out. A portion of the excised tissue was instantly kept in liquid nitrogen. The remainder were dehydrated in 30% sucrose for 24 h after being fixed in 4% paraformaldehyde for 6 h.

### 2.6. Serum Analysis

ELISA was employed on rat serum samples using some ELISA Kits. Serum aspartate aminotransferase (AST), alanine aminotransferase (ALT), alkaline phosphatase (ALP), total bile acid (TBA), albumin (ALB), and γ-glutamyltransferase (γ-GT) levels were measured by using a Chemray 800 automatic analyzer (Hitachi High-Tech, Tokyo, Japan) provided by Sevier Biology.

### 2.7. Histologic and Immunohistochemical Analyses

Briefly, 4–6 μm sections of preserved liver tissues were prepared and stained with H&E or immunofluorescent antibodies. A pathologist with experience in hepatology used an Olympus BX41 microscope (Pannoramic 250 Flash III scanner) to image, examine, and score liver tissue sections in a blinded way.

Immunohistochemical analyses were performed with sham group samples and AAV8-Luc and AAV8-Il18bp liver grafts. The primary antibody was rabbit anti-CD56 monoclonal antibody (14255-1-AP, proteintech, Wuhan, China) (1:2000 dilution; 120 min at room temperature). HRP goat anti-rabbit antibody was used as the secondary antibody for 60 min at room temperature (31460, 1:1600 dilution, Invitrogen, Carlsbad, CA, USA). All histologic analyses were performed by Image-Pro Plus (IPP).

### 2.8. Quantitative Real-Time PCR (qRT-PCR) Analysis

The AG RNAex Pro Reagent (AG21101, Accurate, China) was used to extract total RNA from liver tissue. Prior to usage, the RNA was stored at −80 °C in DEPC-treated water. The cDNA samples were synthesized with PrimeScript^TM^ RT Master Mix Kit (Takara, Tokyo, Japan). A SYBR^®^ Premix Ex Taq TM II (Takara, Tokyo, Japan) was utilized to gauge the levels of the genes associated. A total of 5 µL of SYBR Premix, 2 µL of RNA-free water, 0.5 µL of forward and reverse primers, and 2 µL of cDNA made up the reaction mixture. The relative Ct (2^−ΔΔCt^) method was used to determine the relative expression of mRNA between samples, which were then normalized to the rat GAPDH. The specific primers shown in Table 1 were used to amplify the genes.

### 2.9. Western Blotting Analysis

Collected liver tissue was homogenized on ice with RIPA (P0013B, Beyotime technology, Shanghai, China) containing 10% protease inhibitor cocktail (Roche, Mannheim, Germany), phosphatase inhibitor cocktail III (C0004, TargetMol, Boston, MA, USA) to extract the total proteins. A Pierce™ BCA Protein Assay Kit (Thermo Fisher Scientific, Waltham, MA, USA) was used to quantify the concentrations of the proteins according to the standard instructions. Each protein sample (15 μg) underwent 12% SDS-PAGE separation and was transferred onto polyvinylidene fluoride (PVDF) membranes (Millipore, Billerica, MA, USA). Then, the blots were blocked with 5% skim milk for 2 h at room temperature and incubated 10–12 h at 4 °C with the following primary antibodies shown in Table 2.

Then, the membranes were incubated with goat anti-rabbit or anti-mouse horseradish peroxidase-conjugated secondary antibodies (1:5000 dilution, Santa Cruz Biotechnology) at room temperature for 2 h. The signals were visualized with ECL detection reagents (Millipore, Billerica, MA, USA). The relative expression of the protein was determined by using a ChemiDoc™ XRS+ System and quantified by Image Lab v.4.0 software (Bio-Rad, Hercules, CA, USA).

### 2.10. Statistical Analysis

We carried out the statistical analyses through SPSS 22.0 software. The mean and standard error of the mean (SEM) are displayed for all data, and significance was determined using several tests. For the statistical analysis of three groups, one-factor analysis of variance (ANOVA) was performed. Using Kaplan–Meier curves, the survival periods of recipients in different groups were compared. For each condition investigated, triplicate measurements were included in each experiment. *p* < 0.05 and *p* < 0.01 were considered statistically significant.

## 3. Results

### 3.1. AAV8-Il18bp Treatment Caused Specific IL-18BP Overexpression in the Liver of LEW Rats

**Figure 1:**
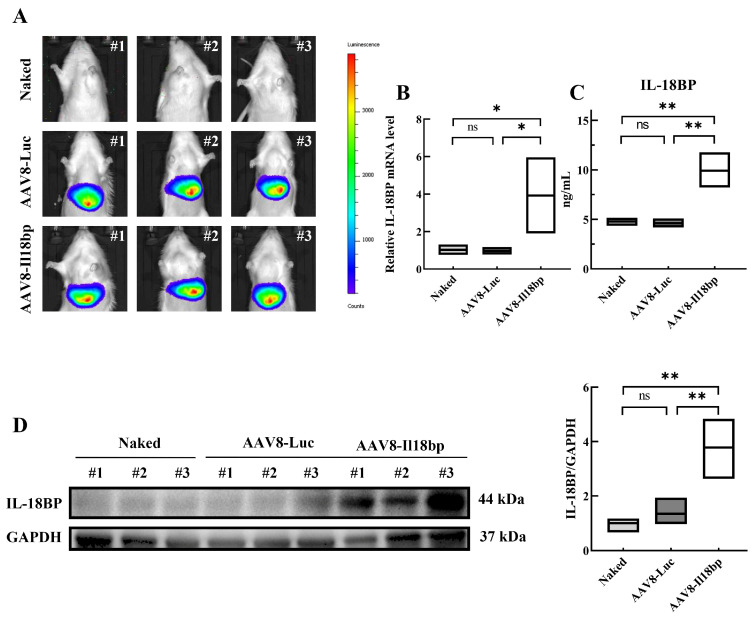
Specific overexpression of IL-18BP in the liver of LEW rats treated with AAV8-Il18bp. (**A**) Live imaging of animals showed that the livers of LEW rats administered with AAV8-Luc and AAV8-Il18bp emitted significant bioluminescence after intraperitoneal injection of fluorescein potassium salt; however, naked LEW rats did not emit bioluminescence. (**B**) The mRNA levels of IL-18BP were significantly increased in the liver tissue of LEW rats given AAV8-Il18bp compared with LEW rats given AAV8-Luc or untreated. (**C**) ELISA showed serum IL-18BP levels were higher in the AAV8-Il18bp group than in either of the other two groups. (**D**) Western blotting results showed that the livers of LEW rats administered with AAV8-Il18bp could significantly express IL-18BP compared with the other groups. Results represent the mean ± SEM (*n* = 3 per group); * *p* < 0.05; ** *p* < 0.01.

Untreated LEW rats and LEW rats injected with 200 µL of AAV8-Luc or AAV8-Il18bp at a concentration of 1 × 10^12^ vg/mL were fed for three weeks. Living animal imaging showed that the livers of the naked rats did not fluoresce, but the livers of the rats that could decompose biotin fluoresced in the presence of Luciferase after caudal vein injection of AAV-8 (Figure 1A). The results showed that AAV-8 could specifically infect rat livers and overexpress the genes carried by AAV-8. From untreated LEW rats and LEW rats injected with AAV8-Luc or AAV8-Il18bp, liver tissue samples were obtained. The mRNA expression was analyzed by qRT-PCR, protein in tissues was detected using Western blotting, and serum IL-18BP levels were assessed through ELISA. In comparison to the other two groups, the AAV8-Il18bp group had greater levels of IL-18BP mRNA (*p* < 0.05) (Figure 1B). Additionally, Western blotting research revealed that the AAV8-Il18bp group’s liver tissue had larger quantities of IL-18BP protein (*p* < 0.01) (Figure 1D). Additionally, the AAV8-Il18bp group had higher serum IL-18BP levels (*p* < 0.05) (Figure 1C).

### 3.2. Levels of Inflammatory Factors in Groups with Different Treatment

The inflammatory reaction is the major manifestation of rejection after liver transplantation. Inflammatory factors secreted by inflammatory cells, such as IFN-γ, IL-1β, TNF-α, IL-6, and IL-8, play important roles in rejection by increasing vascular permeability, enhancing the adhesion of leukocytes, and damaging normal tissues through the infiltration of inflammatory cells. It is widely recognized that inflammation plays a role in the mechanism of immunological rejection through a complex interaction between cells and several soluble cytokines. In the early phase of the inflammatory response after liver transplantation, multiple factors can induce COX-2 expression [34], and of course, ischemia-reperfusion also promotes prostaglandins (PGs) to be continuously released, inducing platelet and neutrophil aggregation, leading to vasoconstriction, and increasing microvascular permeability [35,36]. The persistent production of COX-2 in pathological conditions causes the release of downstream inflammatory factors and promotes the aggravation of inflammation. Interestingly, it was discovered that COX-2 inhibition prevents ischemia-reperfusion injury in organs including the liver [37,38].

**Figure 2:**
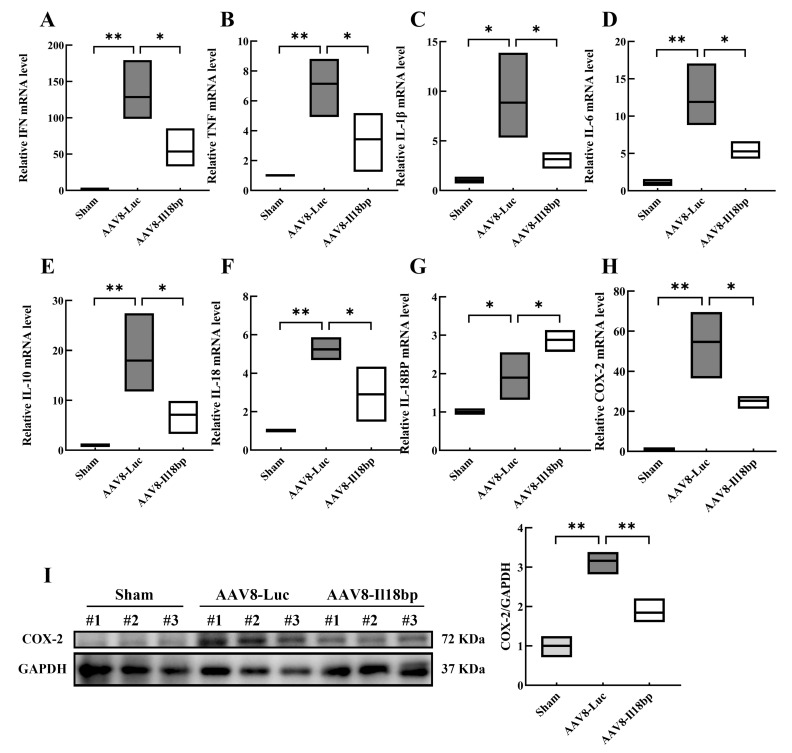
mRNA levels of inflammatory factors in different groups. (**A**–**E**) Compared with the sham group, levels of IFN, TNF, IL-1β, IL-6, and IL-10 mRNA were significantly increased in allografts after donor pretreatment with AAV8-Luc. However, levels of IFN, TNF, IL-1β, IL-6, and IL-10 mRNA were significantly lower in allografts after donor pretreatment with AAV8-Il18bp. (**F**) The level of IL-18 mRNA was significantly increased after OLTx with AAV8-Luc, and infection of AAV8-Il18bp could decrease the IL-18 mRNA level. (**G**) The level of IL-18BP mRNA was significantly increased after OLTx with AAV8-Luc, and infection of AAV8-Il18bp could also increase the IL-18BP mRNA level. (**H**) The level of COX-2 mRNA was analyzed by qRT-PCR. (**I**) Western blotting analysis of COX-2 in liver tissue after sham or OLTx in different groups. Results represent the mean ± SEM (*n* = 3 per group); * *p* < 0.05; ** *p* < 0.01.

Seven days following OLTx, total RNA from liver allografts was extracted and subjected to qRT-PCR analysis. When compared to the sham group, the allografts had significantly higher mRNA levels of IFN, TNF, IL-1β, IL-6, and IL-10. Additionally, allografts from donors who had received pretreatment with AAV8-Il18bp had significantly decreased mRNA levels of IFN, TNF, IL-1, IL-6, and IL-10 (Figure 2A–E).

**Figure 3:**
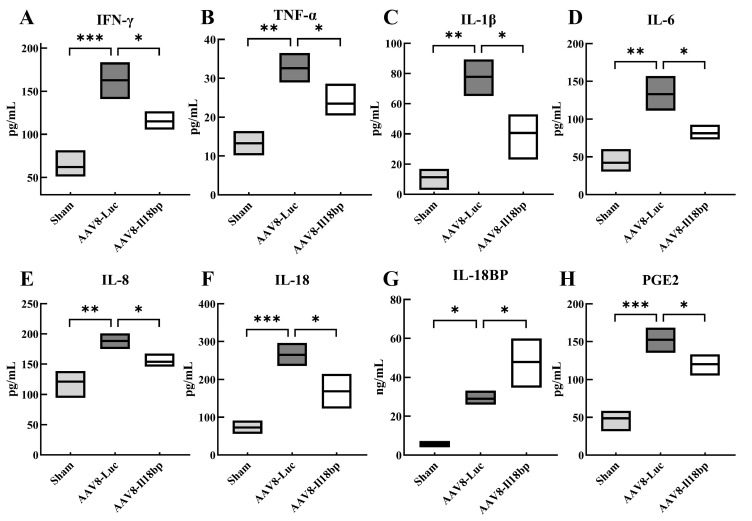
Serum levels of inflammatory factors in different groups. (**A**–**E**) Serum levels of IFN-γ, TNF-α, IL-1β, IL-6, and IL-8 in recipients whose donors were pretreated with AAV8-Luc were significantly higher than those in the sham group. However, these inflammatory factors in recipients whose donors were pretreated with AAV8-Il18bp were significantly lower than those in recipients whose donors were given AAV8-Luc. (**F**) The level of IL-18 was significantly increased after OLTx with AAV8-Luc, and infection of AAV8-Il18bp could decrease the IL-18 level in serum. (**G**) Serum level of IL-18BP was significantly increased after OLTx with AAV8-Luc. (**H**) Serum level of PGE2 was detected by ELISA. Results represent the mean ± SEM (*n* = 3 per group); * *p* < 0.05; ** *p* < 0.01; *** *p* < 0.001.

The secretion of inflammatory factors in recipient serum was examined by ELISA. Serum IFN-γ, TNF-α, IL-1β, IL-6, and IL-8 levels in recipients whose donors had received pretreatment with AAV8-Luc were significantly higher than the sham group, as shown in Figure 3A–E. Additionally, in recipients whose donors had received pretreatment with AAV8-Il18bp, serum levels of these cytokines were substantially lower (Figure 3A–E). These results showed that infection with AAV8-Il18bp significantly decreased inflammatory factor expression in recipients after OLTx.

Furthermore, following OLTx with AAV8-Luc, the amount of IL-18 mRNA was considerably higher (*p* < 0.01) and infection of AAV8-Il18bp could decrease the IL-18 mRNA level (*p* < 0.05) (Figure 2F). After OLTx with AAV8-Luc, IL-18BP mRNA levels considerably increased. However, infection of AAV8-Il18bp could increase the IL-18BP mRNA level more than the AAV8-Luc group (*p* < 0.05) (Figure 2G). In addition, serum IL-18 was significantly increased after OLTx with AAV8-Luc (*p* < 0.001), and infection of AAV8-Il18bp could decrease the IL-18 level (*p* < 0.05) (Figure 3F). The serum level of IL-18BP was considerably increased after OLTx with AAV8-Luc (*p* < 0.05). A higher level of serum IL-18BP was shown in the AAV8-Il18bp group (*p* < 0.05) (Figure 3G). Our findings indicated that IL-18BP could reduce inflammatory reactions after transplantation.

We also conducted qRT-PCR and Western blotting to detect the expression of COX-2 mRNA and protein. Compared with the sham group, the content of COX-2 mRNA and protein remarkably increased after OLTx of the AAV8-Luc group (*p* < 0.05). However, compared with the AAV8-Luc group, intervening with AAV8-Il18bp reduced the expression of COX-2 (*p* < 0.05) (Figure 2H, I). What is more, an ELISA assay was performed to detect the expression levels of PGE2 (the downstream product of COX-2) in serum. As shown in Figure 3H, compared with the sham group, the content of PGE2 in the AAV8-Luc group was significantly increased (*p* < 0.001). Conversely, in the AAV8-Il18bp group, the level of PGE2 was down-regulated (*p* < 0.05).

### 3.3. Increased IL-18BP Levels Protected Liver Function from Immune Rejection after LEW-BN OLTx

**Figure 4:**
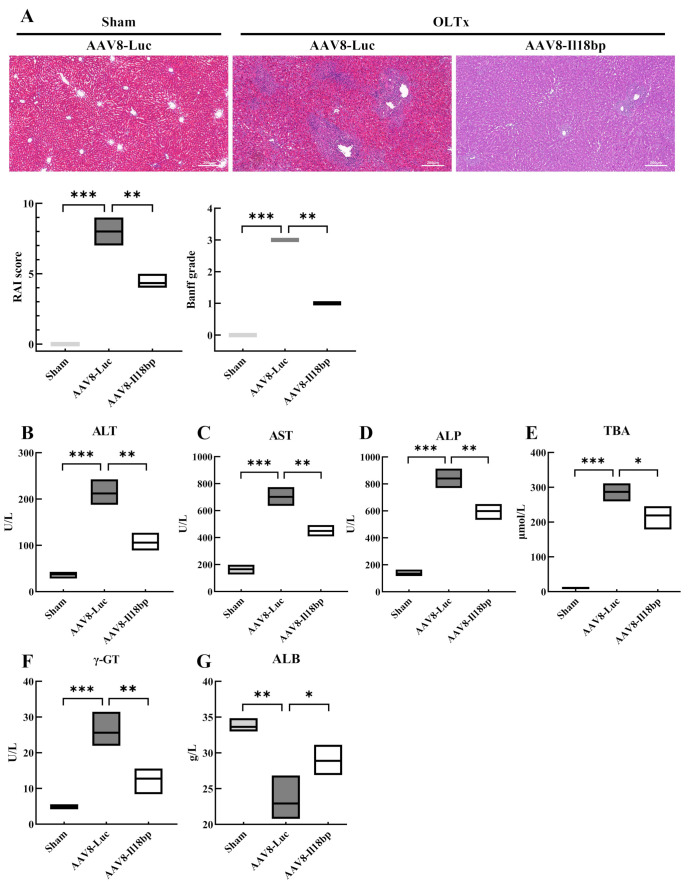
Increased IL-18BP levels protected liver function from immune rejection after LEW-BN OLTx. (**A**) Histopathological changes in the liver after LEW-BN OLTx were shown by HE staining. Liver histopathologic analysis RAI score for Banff criteria (sham group: 0 points; AAV8-Luc group: 7–9 points; AAV8-Il18bp group: 3–5 points). Banff grade for Banff criteria (sham group: grade 0; AAV8-Luc group: grade III; AAV8-Il18bp group: grade I). Scale bar = 200 μm. (**B**–**F**) After OLTx, serum ALT, AST, ALP, TBA, and γ-GT levels were significantly increased. Those indicators in recipients whose donors were pretreated with AAV8-Il18bp were significantly lower than those in recipients whose donors were given AAV8-Luc. (**G**) Serum ALB levels in recipients whose donors were pretreated with AAV8-Il18bp were a little increased. All indicators of liver function in the sham group were within the normal range. Results represent the mean ± SEM (*n* = 3 per group); * *p* < 0.05; ** *p* < 0.01; *** *p* < 0.001.

Graft survival time after liver transplantation is a determinant of the recipient’s survival time, and protecting the graft from attack by the recipient’s immune system can significantly prolong its survival. As shown in Figure 4A, histopathologic analysis illustrated that livers from the sham group displayed no significant immune rejection or pathological damage. The hepatic tissue structure was normal, with hepatocytes arranged radially along the central vein, forming hepatic cords, without degeneration or necrosis; the hepatic sinusoids were not dilated and congested; no pathological changes such as bile duct injury and immune cell infiltration were seen in the confluent area. However, in the AAV8-Luc group, scattered or focal necrosis of hepatocytes and hyperplasia of blastocytes in the hepatic sinusoids were seen on the 7th day after transplantation; massive mixed inflammatory cell infiltrations in the confluent area, involving the bile ducts and blood vessels, with bile duct degeneration, inflammatory cell infiltration, and necrosis under the intima of the hepatic veins were also seen, which was a rejection reaction grade of III (7–9 points). In contrast to the AAV8-Luc group, the infiltration of inflammatory cells, such as lymphocytes, in the confluent region was dramatically decreased in the AAV8-Il18bp group; there was only partial inflammatory damage to the bile ducts, and the degree of phlebitis was less severe, which was a rejection reaction grade of I (3–5 points).

What is more, the liver engages in important physiological functions, such as metabolism, detoxification, and synthesis, and the indicators of liver function can reflect the basic status of the liver. Serological analysis of the recipients was also performed. Tests of liver function were utilized to assess the severity of liver damage, and serum levels of ALT, AST, ALP, TBA, γ-GT, and ALB were measured in BN recipients who received livers from LEW rats 7 d after OLTx.

Apart from histological changes, increases in ALT, AST, ALP, and γ-GT indicate inflammation in the liver and significant liver damage. ALB is synthesized by the liver, and when liver function is impaired, albumin synthesis is reduced. Elevated TBIL, DBIL, and TBA indicate that the liver is excreting bile, and TBA is a more sensitive indicator of the excretory function of the liver than TBIL or DBIL.

As shown in Figure 4B–F, serum ALT, AST, ALP, TBA, and γ-GT were significantly higher in recipients whose donors had received pretreatment with AAV8-Luc compared to the sham group. Additionally, recipients whose donors had previously received AAV8-Il18bp pretreatment had lower ALT, AST, ALP, TBA, and γ-GT levels than the AAV8-Luc group. In comparison to the sham group, serum ALB levels in the AAV8-Luc group were considerably lower. Moreover, compared to the AAV8-Luc group, serum ALB levels in recipients whose donors had received pretreatment with AAV8-Il18bp were marginally higher. (Figure 4G). The results of both histological and biochemical tests indicated that AAV8-Il18bp had the ability to attenuate the extent of liver tissue damage after transplantation.

### 3.4. Elevated IL-18BP Levels Protected the Liver from Apoptosis after LEW-BN OLTx

**Figure 5:**
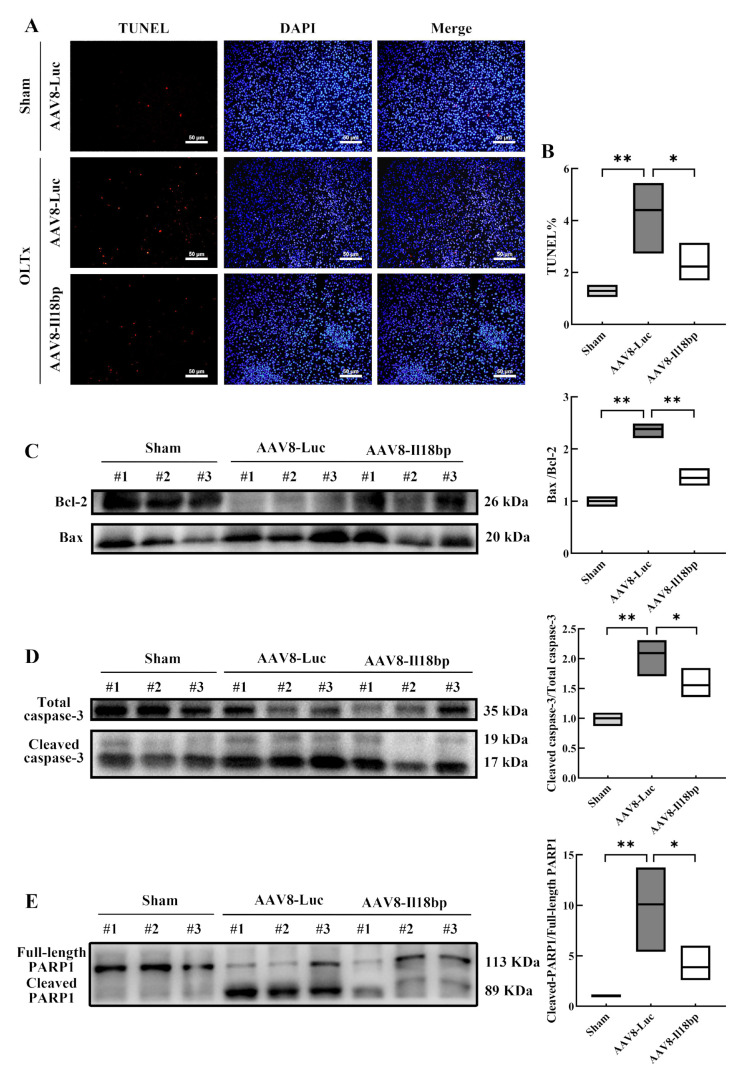
Elevated IL-18BP levels protected the liver from apoptosis after LEW-BN OLTx. (**A**) TUNEL staining was performed to assess the apoptosis rate. Representative photomicrograph of TUNEL-positive staining (red) in the liver in different groups (scale bar = 50 μm). (**B**) Ratios of TUNEL-positive cells in the liver in different groups. (**C**) Bax and Bcl-2 protein expression in different groups were analyzed by Western blotting. (**D**) Total caspase-3 and cleaved caspase-3 protein expression in different groups were analyzed by Western blotting. (**E**) Full-length PARP1 and cleaved PARP1 protein expression in different groups were analyzed by Western blotting. TUNEL: terminal deoxynucleotidyl transferase biotin-dUTP nick end labeling. Results represent the mean ± SEM (*n* = 3 per group); * *p* < 0.05; ** *p* < 0.01.

To further determine the role of IL-18BP overexpression in inhibiting hepatocyte apoptosis, the TUNEL assay was carried out to measure the rate of hepatocyte apoptosis following OLTx. The results indicated that the sham group injected with the AAV8-Luc had only a few TUNEL-positive (red fluorescent) cells, and the frequency of apoptosis was 1.29% ± 0.11%. However, in the AAV8-Luc group, numerous TUNEL-positive cells were seen after OLTx, and the frequency was 4.40% ± 0.69% (*p* < 0.01). Distinct from the AAV8-Luc group, the TUNEL-positive cells were 2.23% ± 0.38% and were observably reduced in the AAV8-Il18bp group after transplantation (*p* < 0.05) (Figure 5A,B). The TUNEL assay results demonstrated that upregulation of IL-18BP decreased hepatocyte apoptosis and might shield the graft from immune system rejection in the recipient. Consistent with the TUNEL assay results, agarose gel electrophoresis analysis similarly indicated that IL-18BP was effective in protecting liver tissue from apoptosis after OLTx (Figure S1).

In addition, apoptosis-related protein expression in liver tissue was also assessed, including Bax, Bcl-2, cleaved caspase-3, and cleaved PARP1. As shown in Figure 5C, after LEW-BN OLTx, the ratio of Bax/Bcl-2 in the liver was raised compared with the control group (*p* < 0.01). After AAV8-Il18bp transfection, the ratio of Bax/Bcl-2 was obviously decreased (*p* < 0.01). To evaluate the expression of both total and cleaved caspase-3, full-length PARP1 and cleaved PARP1, Western blotting analysis was also carried out. We discovered that OLTx without AAV8-Il18bp dramatically raised the production of cleaved caspase-3 and cleaved PARP1 in hepatic tissue (*p* < 0.01). When IL-18BP was overexpressed, the expression of cleaved caspase-3 and cleaved PARP1 reduced (*p* < 0.05) (Figure 5D, E). Consistent with the TUNEL assay results, Western blotting analysis similarly indicated that IL-18BP was effective in protecting liver tissue from apoptosis after OLTx.

### 3.5. IL-18BP Overexpression Reduced CD56 Expression after LEW-BN OLTx

**Figure 6:**
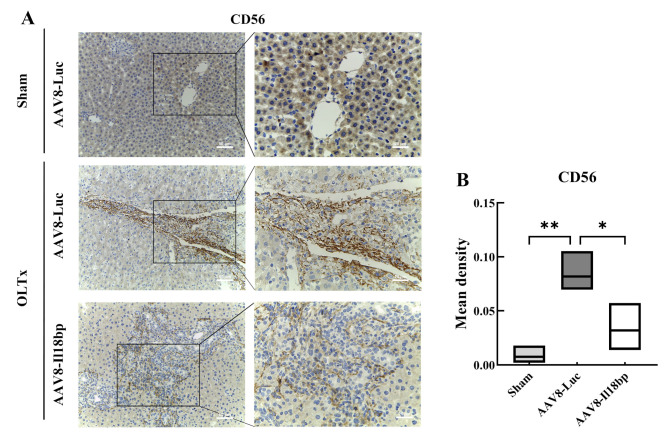
IL-18BP overexpression reduced CD56 expression after LEW-BN OLTx. (**A**) Representative images of IHC staining for CD56 protein in livers of different groups. Scale bar, 50 μm, 25 μm. (**B**) The cumulative frequency of tissue-infiltrating CD56^+^NK cells detected by IPP 6.0 in each group; error bars represent means ± SEM (*n* = 3 per group); * *p* < 0.05; ** *p* < 0.01.

The expression of CD56 (NCAM) in diseased livers has been described on BDC with high levels of hepatic necrosis, FNH, and chronic cholestatic diseases but was absent on BECs in normal livers [39,40]. These studies suggest that CD56 (NCAM) may serve as a useful marker of the repair or regeneration status of biliary tract cells in different liver diseases [41]. As shown in Figure 6, when the AAV8-Luc group underwent OLTx, we observed high expression of CD56 (NCAM) around the biliary line in liver tissue. However, IL-18BP overexpression could notably reduce the expression of CD56 (NCAM) (*p* < 0.05). Considering these results, we believe that CD56 (NCAM) has a positive correlation with the degree of liver injury, the occurrence of immune rejection in the graft, and the presence of IL-18 in serum. Hence, we suggest that IL-18 is crucial for activating NK cells against grafts and that immune rejection of grafts is attenuated when AAV8-Il18bp enables robust IL-18BP generation to effectively counteract the effects of IL-18.

### 3.6. High Level of IL-18BP Prolonged Survival Time of the Recipients after OLTx

**Figure 7:**
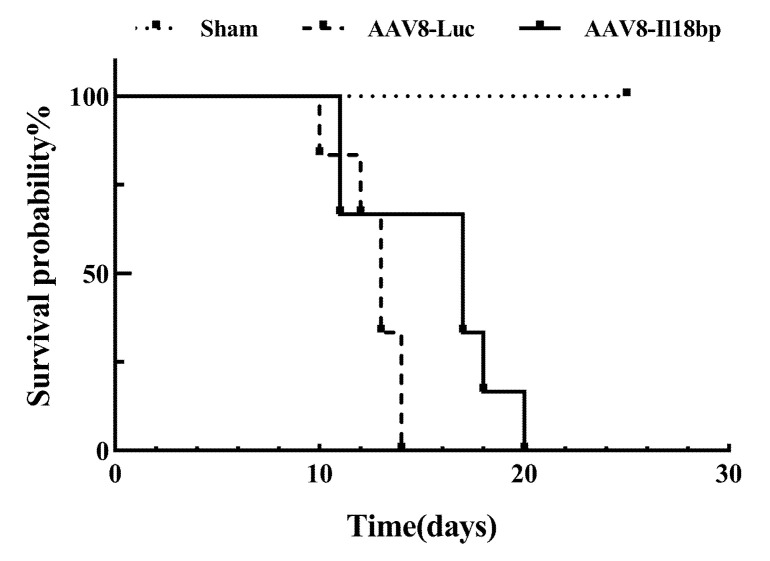
High levels of IL-18BP prolonged survival time of the recipients after OLTx. Recipients whose donors were pretreated with AAV8-Il18bp had significantly prolonged survival compared with those whose donors were given AAV8-Luc. (*n* = 6 in each group, median survival time (MST): 13 vs. 17 d); *p* < 0.01.

To explore the overall benefit of AAV8-Il18bp delivery in LEW-BN OLTx, the survival of recipient animals that underwent OLTx was assessed. Figure 7 illustrates that patients who received liver allografts from donors who had given AAV8-Il18bp pretreatment lived longer than patients whose donors had administered AAV8-Luc (median survival time, 17 versus 13 d) (*p* < 0.01). The results indicate that IL-18BP overexpression not only attenuated acute vascular rejection in grafts but also significantly prolonged the survival time of BN rats after OLTx.

## 4. Discussion

First discovered in Kupffer cells, IL-18 was shown to be an IFN-γ-inducing cytokine that stimulated NK cells in the mouse [13]. When caspase-1 cleaves pro-IL-18 [42], IL-18 can bind to IL-18R [43], which brings about the recruitment and heterodimerization of IL-18RAcP and causes downstream recruitment of NF-κB [44] and AP-1 [45]. The pro-inflammatory properties of IL-18 include its capacity to stimulate the secretion of inflammatory cytokines and chemokines while inhibiting the synthesis of IL-10. Inhibiting the binding of IL-18 to IL-18R, IL-18BP prevents the generation of IFN-γ and reduces the response of NK and Th1 cells. More interestingly, there is a clear negative feedback link between IL-18BP and IL-18. The balance is sustained in vivo by the upregulation of IL-18BP expression caused by IFN-γ production.

The production of IL-18 linked with a histologic change and the stimulation of IFN-γ in a mouse model of heart transplantation. Similarly, our group considered that the abnormal expression of IL-18 and the dysregulation of IL-18BP mediated AVR after pig–NHPs liver xenotransplantation. Based on this underlying mechanism, we explored the protective effect of IL-18BP on AVR.

In earlier research, the function of IL-18 was precisely inhibited by an adenoviral vector Adex-IL18bp, and the impact of this protective effect on the liver was investigated using a rat model of OLTx (ACI to Lewis) [46]. Seven days after OLTx, recipient rats with Adex-IL18BP-prepared donors had significantly lower blood ALT levels and lessened histologic liver damage compared to the Adex-LacZ control group. Additionally, Adex-IL18bp pretreatment dramatically increased rat/allograft survival, suppressed the production of IFN-γ and lowered levels of CXCL10 and CX3CL1 [46]. However, the average survival time of the recipients of Adex-Il18bp-pretreated donor liver grafts was only 13 days, which is approximately the same time that adenovirus can act. Therefore, we hypothesize that the possible cause of recipient death was a decrease in IL-18BP production by the donor liver.

To learn more about the function of IL-18BP in our study, we constructed an AAV-8 vector carrying Il18bp and the liver-specific promoter TBG, which could specifically target liver tissue for infection and could stably express the target gene for more than six months [47,48]. Additionally, we explored the function of IL-18BP in preserving the liver from AVR in the LEW-BN rat OLTx model, which is widely recognized internationally.

Intravital optical images showed that the livers of untreated rats did not fluoresce, while the liver of the rats could decompose biotin in the presence of Luciferase after infection with AAV8 vectors. The results showed that AAV8 could specifically infect the rat liver. Additionally, the AAV8-Il18bp group had greater levels of IL-18BP mRNA, serum IL-18BP, and IL-18BP protein expression than either of the other two groups. These results indicate that AAV8-Il18bp infection can provide adequate IL-18BP expression.

Furthermore, earlier research showed that IFN-γ, which is generated by NK cells immediately after rat OLTx, is an essential modulator in AVR [49]. Nevertheless, early OLTx in AVR involves a network of several inflammatory factors. Therefore, we examined the mRNA expression of not only IFN but also other inflammatory factors including TNF, IL-1β, IL-6, and IL-10. The qRT-PCR results demonstrated that early OLTx lacking the transfection of AAV8-Il18bp had observably higher levels of inflammatory factor mRNA expression. However, livers that had been infected with AAV8-Il18bp for OLTx showed a significant decreased mRNA level of inflammatory cytokines. Additionally, serum levels of IFN-γ, TNF-α, IL-1β, IL-6, and IL-8 were measured in BN recipients of LEW livers 7 d after OLTx. The ELISA results showed approximately the same as qRT–PCR results. What is more, the metabolic pathway of cyclooxygenase (COX) plays a prominent role in the process of immunological rejection. In pathological conditions, the prolonged production of COX-2 leads to the release of downstream inflammatory factors and aids the exacerbation of inflammation. Therefore, we conducted qRT-PCR and Western blotting to detect the expression of COX-2 mRNA and protein, and ELISA to examine the serum level of PGE2. Our results indicated that IL-18BP could alleviate the inflammatory response after transplantation.

Furthermore, patients whose donors received AAV8-Il18bp rather than AAV8-Luc had better liver function, as evidenced by decreased blood ALT, AST, ALP, TBA, and γ-GT levels and higher serum ALB levels at 7 days following OLTx. Histopathologic investigations also supported this protective effect. After pretreatment with AAV8-Il18bp, inflammatory cell infiltration, such as lymphocytes, in the confluent area was significantly reduced, there was only partial inflammatory damage to the bile ducts, and the degree of phlebitis was less severe, which indicated a rejection reaction of grade I. Our experiments confirmed that AAV8-Il18bp attenuated immune rejection of liver tissues and maintained normal physiological functions of the liver. However, the mechanism associated with liver injury is not clear. Following that, we carried out a TUNEL experiment and discovered that after infection with AAV8-Il18bp in contrast to the AAV8-Luc group, the proportion of apoptotic cells in liver tissue sections was much lower. The Western blotting results suggested that hepatocytes underwent apoptosis via activation of the Bax/Bcl-2/cleaved caspase-3/cleaved PARP1 signaling pathway and AAV8-Il18bp may inhibit the production of proteins that promote apoptosis.

Numerous studies have provided evidence for the significance of IL-18 in the pathophysiology of inflammation and the immune response [50,51,52,53,54]. Hideaki Obara [49] discovered NK-cell infiltration in the early stages of liver transplantation, revealing a crucial mechanism for the participation of NK cells in AVR. According to earlier research, IL-18 can interact with NK cells to enhance their ability to activate and kill, and we discovered that the dysregulated IL-18/IL-18BP in pig–NHPs liver xenotransplantation may be connected with the involvement of NK cells in AVR. Our findings demonstrated that a strong and favorable correlation between the ratio of IL-18/IL-18BP and NK-cell infiltration in the paravalvular region and that a persistently high concentration of IL-18BP in the liver protected the graft from rejection, which could be helpful for subsequent gene-edited pigs, as well as clinical xenotransplantation or targeting gene therapy using AAV-8 vector.

Ono et al. [46] also counted the survival time of recipients and showed that recipients that received Adex-Il18bp-pretreated donor liver grafts survived slightly longer than recipients who received Adex-LacZ donors. Therefore, we investigated the survival time of BN rats who received a donor liver transfected with AAV8- Il18bp vectors that could consistently express IL-18BP for more than six months in vivo. Our results indicated that Il18bp gene delivery to liver allografts can extend graft survival time in the LEW-BN rat OLTx model relative to the control group, albeit the increase in survival time was not statistically significant compared to Ono’s research. We speculate that the cause of this phenomenon is the distinction between the two animal models or the enhanced effect of other mediated immune rejection after the inhibition of IL-18. Although we proved that IL-18BP has a protective impact on LEW-BN rat OLTx, it still could not maintain graft survival for a long time. Thus, the deeper mechanisms need to be further investigated so that we can alleviate the problem of insufficient donor numbers.

## Figures and Tables

**Table 1:** Sequence of primers involved in this study.

Gene (Rat)	Forward Primer	Reverse Primer
GAPDH	GCTGAGTATGTCGTGGAGTCTAC	TTCCACGATGCCAAAGTTGTCAT
IL-18BP	CAGAGAAAGAAGTGCCACTGAATG	GGATCCACAAACAGACAGGAGAA
IL-18	TCGTAAACCAGGCTTTCACTTCT	CAGGCAGCTAGGATTACGGAAAG
IFN	GCACACTCATTGAAAGCCTAGAA	AGGTGCGATTCGATGACACTTAT
TNF	GCACACTCATTGAAAGCCTAGAA	CCTTGAAGAGAACCTGGGAGTAG
IL-1β	TGCTAGTGTGTGATGTTCCCATTA	GTCGTTGCTTGTCTCTCCTTGTA
IL-6	TTCCGTTTCTACCTGGAGTTTGT	TTAGGAGAGCATTGGAAGTTGGG
IL-10	GTGACAATAACTGCACCCACTTC	GGGCATCACTTCTACCAGGTAAA
COX-2	CTTCCTCCTGTGGCTGATGACTG	GGTCCTCGCTTCTGATCTGTCTTG

**Table 2:** Antibodies used in Western blotting.

Western Blotting Antibody	Company	Dilution
IL-18BP	Abcam (ab52914)	1:5000
Bax	Proteintech Group (60267-1-Ig)	1:5000
Bcl-2	Proteintech Group (60178-1-Ig)	1:1000
Caspase-3	Proteintech Group (66470-2-Ig)	1:1000
Cleaved caspase-3	Proteintech Group (66470-2-Ig)	1:1000
COX-2	Proteintech Group (66351-1-Ig)	1:1000
PARP1	Proteintech Group (66520-1-Ig)	1:5000
Cleaved PARP1	Proteintech Group (66520-1-Ig)	1:5000
GAPDH	Proteintech Group (10494-1-AP)	1:10,000

## Data Availability

The data underlying this article will be shared on reasonable request to the corresponding author.

## Supplementary Materials

The following supporting information can be downloaded at: https://www.mdpi.com/article/10.3390/biom12121801/s1, Figure S1: The emergence of DNA fragments was detected through agarose gel electrophoresis.
