# Supplementary material for: IL-18BP Improves Early Graft Function and Survival in Lewis–Brown Norway Rat Orthotopic Liver Transplantation Model
Source: Biomolecules. 2022 Dec 1;12(12):1801. doi: 10.3390/biom12121801 (PMC9775331; doi:10.3390/biom12121801)
Supplement: Supplementary file 1 [file biomolecules-12-01801-s001.zip › biomolecules-2037140-supplementary.pdf]

It is well known that inflammation as a complex interaction between some soluble cytokines and cells participates in the process of immunological rejection. The metabolic pathway of cyclooxygenase (COX) plays a prominent role in this process. The COX-catalyzed conversion of AA to prostaglandins (PGS) and other inflammatory mediators encourage the inflammatory response. In pathological situations, the continued production of COX-2 triggers the release of downstream inflammatory factors and facilitates the aggravation of inflammation.
Therefore, we conducted qRT-PCR and western blotting to detect the expression of COX-2 mRNA and protein. Compared with sham group, the content of COX-2 mRNA and protein remarkably increased after OLTx of AAV8-Luc group; However, compared with the AAV8-Luc group, intervening with AAV8-Il18bp reduced the expression of COX-2 (Figure S1A,B).
We also performed ELISA assay to detect the expression levels of PGE2 (the downstream product of COX-2) in serum. As shown in Figure S1C, compared with the sham group, the content of PGE2 in AAV8-Luc group was significantly increased. Conversely, in AAV8-Il18bp group, the level of PGE2 was down-regulated.

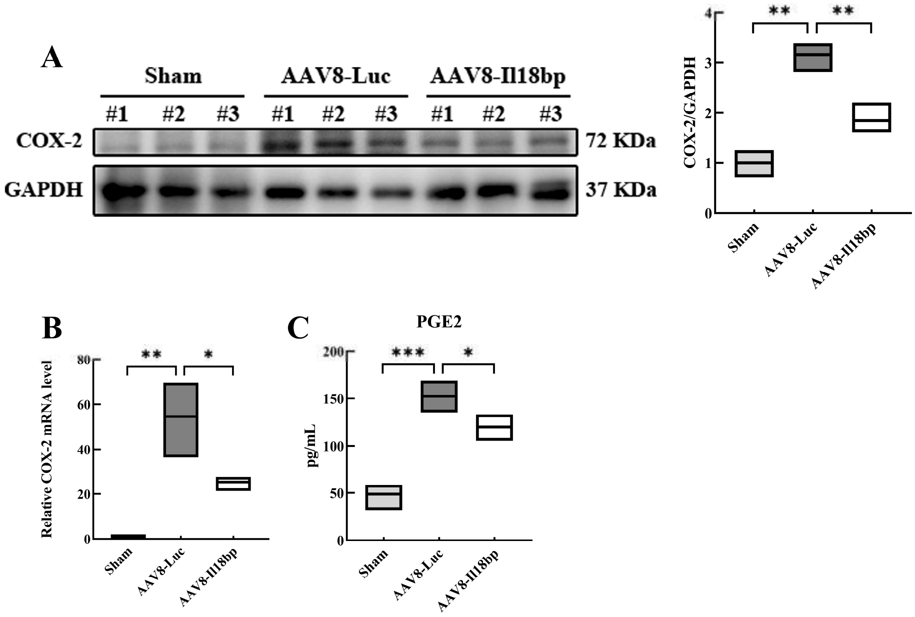

Figure S1. The emergence of DNA fragments was detected through agarose gel electrophoresis. * $p < 0.05$; ** $p < 0.01$; *** $p < 0.001$.
